# BioLemmatizer: a lemmatization tool for morphological processing of biomedical text

**DOI:** 10.1186/2041-1480-3-3

**Published:** 2012-04-01

**Authors:** Haibin Liu, Tom Christiansen, William A Baumgartner, Karin Verspoor

**Affiliations:** 1 Colorado Computational Pharmacology, University of Colorado School of Medicine, Aurora, CO 80045, USA; 2 National ICT Australia, Victoria Research Lab, Melbourne 3010, Australia

## Abstract

**Background:**

The wide variety of morphological variants of domain-specific technical terms contributes to the complexity of performing natural language processing of the scientific literature related to molecular biology. For morphological analysis of these texts, lemmatization has been actively applied in the recent biomedical research.

**Results:**

In this work, we developed a domain-specific lemmatization tool, BioLemmatizer, for the morphological analysis of biomedical literature. The tool focuses on the inflectional morphology of English and is based on the general English lemmatization tool MorphAdorner. The BioLemmatizer is further tailored to the biological domain through incorporation of several published lexical resources. It retrieves lemmas based on the use of a word lexicon, and defines a set of rules that transform a word to a lemma if it is not encountered in the lexicon. An innovative aspect of the BioLemmatizer is the use of a hierarchical strategy for searching the lexicon, which enables the discovery of the correct lemma even if the input Part-of-Speech information is inaccurate. The BioLemmatizer achieves an accuracy of 97.5% in lemmatizing an evaluation set prepared from the CRAFT corpus, a collection of full-text biomedical articles, and an accuracy of 97.6% on the *LLL05 *corpus. The contribution of the BioLemmatizer to accuracy improvement of a practical information extraction task is further demonstrated when it is used as a component in a biomedical text mining system.

**Conclusions:**

The BioLemmatizer outperforms other tools when compared with eight existing lemmatizers. The BioLemmatizer is released as an open source software and can be downloaded from *http://biolemmatizer.sourceforge.net*.

## Background

An important fundamental natural language processing (NLP) task is lemmatization. Lemmatization is a morphological transformation that changes a word as it appears in running text into the base or dictionary form of the word, which is known as a lemma, by removing the inflectional ending of the word. The lemma corresponds to the singular form in the case of a noun, the infinitive form in the case of a verb, and the positive form in the case of an adjective or adverb. We can think of lemmatization as a normalization process in which different morphological variants of a word are mapped into the same underlying lemma so they can be analyzed as a single item (term or concept). By reducing the total number of distinct terms, lemmatization decreases the complexity of the analyzed text, and therefore brings important benefits to downstream text processing components. For instance, when incorporated in an information retrieval system, lemmatization can help to improve overall retrieval recall since a query will be able to match more documents when variants in both query and documents are morphologically normalized [1]. Similarly, natural language understanding systems are able to work with linguistically normalized terms, effectively semantic concepts, rather than having to individually handle all surface variants of a word [2].

Stemming, another word form normalization technique, has also been widely applied in information retrieval [3]. Stemming normalizes several morphological variants of a word into the same form, known as a stem, by stripping off the suffix of a word. Though the goals of stemming are similar to those of lemmatization, an important distinction is that stemming does not aim to generate a naturally occurring, dictionary form of a word - for instance, the stem of "regulated" would be "regul" rather than the base verb form "regulate". This often results in incorrect conflation of semantically distinct terms [4]. For instance, the terms "activates", "activations" and "activities" would all be (over-) stemmed to "activ" or "act" by most stemming algorithms [5,6], while a lemmatizer would treat them as having distinct base forms (note that "activates" and "activation" will be maintained as distinct for a lemmatizer that handles only inflectional morphology since the former is a verb form and the latter a noun). On the other hand, existing stemming algorithms may not correctly conflate related inflected forms, such as "actor" and "action" (understemming). Compared to the truncated, ambiguous stems that stemming often returns, more linguistically-based lemmas have shown advantages in document clustering and information extraction [7-9].

The scientific literature related to molecular biology contains a huge number of domain-specific technical terms [10]. In addition to the characteristics of the terms themselves such as Greek letters, digits and other symbols, the wide variety of orthographic and morphological variants of these terms also contributes to the complexity of processing biological literature. For morphological analysis of these texts, lemmatization has been actively applied in the recent biomedical research [2,11,12]. In order to assist in efficient medical text analysis, lemmas rather than full word forms in input texts are often used as a feature for machine learning methods that detect medical entities [11]. Methods that take advantage of syntactic dependency paths to detect mentions of protein-protein interactions in the biomedical scientific literature often utilize lemmatized tokens rather than inflected forms [2,12].

In the more recent BioNLP'11 shared task on event extraction [13], four out of the top nine performing systems of the GENIA event task contain a lemmatization component [9,14-16]. It is demonstrated that the performance of the event extraction system is significantly improved by abstracting literal words to their lemmas [9]. However, all the lemmatization tools used in the shared task, such as *morpha *[17], and WordNet-based [18] lemmatizers, were developed and parameterized only for the general English language, and therefore cannot correctly produce the lemmas for many biomedical terms [9,19,20]. For instance, the domain-specific terms "phosphorylation" and "methylation" are not recorded in the general English thesaurus WordNet. Therefore, applying these tools to the biology domain results in some loss in performance.

The lexical programs using the Specialist lexicon^a ^[21], one of the UMLS knowledge sources at the National Library of Medicine [22], have been used to normalize words in biological texts to account for spelling variations and also to provide lemmas [12]. The Specialist lexicon includes both general English lexical items as well as terms specific to biomedicine, selected from a variety of sources including MEDLINE citations [23], the UMLS Metathesaurus [22], and more than eight medical and general English dictionaries. Although the Specialist lexicon provides a broad coverage of the general biomedical language, it fails to cover in detail the various subdomains of molecular biology, such as gene and protein names [10]. Furthermore, the lexical programs are designed to normalize a word into a form that maps to an entry in the UMLS Metathesaurus in order to facilitate the subsequent semantic analysis. Therefore, in addition to common morphological processing, the normalization process also involves ignoring punctuation, removing genitive markers, ignoring word order, etc. [21]. Therefore, the resulting normalized form may not correspond to the lemma a user expects.

In this work, we developed a domain-specific lemmatization tool, BioLemmatizer, for the morphological analysis of biomedical literature. The BioLemmatizer is based on the general English lemmatizer from the MorphAdorner toolkit [24], and is tailored to the biological domain through integration of several published lexical resources related to molecular biology. It focuses on the inflectional morphology of English, including the plural form of nouns, the conjugations of verbs, and the comparative and superlative form of adjectives and adverbs. Given a word and its Part-of-Speech (POS) usage, the BioLemmatizer retrieves the lemma based on the use of a lexicon that covers an exhaustive list of inflected word forms and their corresponding lemmas in both general English and the biomedical domain, as well as a set of rules that generalize morphological transformations to heuristically handle words not encountered in the lexicon.

Derivational morphology links forms of lexical items grammatically related by affixation, but involve a change in syntactic category [21]. For instance, "malaria" is a noun derivationally related to the adjective "malarial" by the suffix "al". Although the BioLemmatizer natively transforms adverbs to their grammatically related adjectives based on that functionality in the underlying MorphAdorner lemmatizer, it does not currently address derivational morphology for other parts of speech, such as relating nominalizations to their source verbs, or adjectives to their source nouns.

The BioLemmatizer is developed in Java and has been integrated into the Apache Unstructured Information Management Architecture (UIMA) [25]. It is freely available to the NLP and text mining research communities, and is released as open source software that can be downloaded via *http://biolemmatizer.sourceforge.net*. The BioLemmatizer has been successfully applied for quality assurance of the CRAFT corpus [26,27] in preparation for its upcoming public release.

The rest of the paper is organized as follows: First, we review eight different existing tools that all provide a lemmatization function. Then, we report a thorough evaluation of the BioLemmatizer on three different biomedical datasets in comparison with the existing tools. A successful application of the BioLemmatizer is also described in detail. Further, the contribution of the BioLemmatizer to accuracy improvement of an information extraction task is demonstrated. Next, we elaborate the methods and resources used in the BioLemmatizer tool. Finally, we summarize the paper and introduce future work.

### Related work

A number of tools have been developed over the years which provide lemmatization functionality. Despite the different processing techniques employed, all of them make use of a word lexicon, a set of rules, or a combination of the two as the resources for morphological analysis. In this section, we review eight different lemmatization tools, including the WordNet lemmatizer [28], the CLEAR morphological analyzer [29], the GENIA tagger [30], TreeTagger [31], Norm [32], LuiNorm [33], MorphAdorner [24] and *morpha *[17]. The performance of each tool is evaluated thoroughly on biomedical texts in the Results section in order to compare with that of the BioLemmatizer.

The WordNet lemmatizer [28] uses the internal lemmatization algorithm of WordNet [18] to normalize words. The algorithm makes use of two resources, a set of rules which specify the inflectional endings that can be detached from individual words, and a list of exceptions for irregular word forms. It first checks for exceptions and then applies the rules of detachment. After each transformation by rules, the WordNet database is searched for the existence of the resulting form. In our experiments, we slightly modified the standard WordNet lemmatizer to produce lemmas for each input word together with its Part-of-Speech tag. If it fails, the lemmatizer attempts to retrieve all valid WordNet lemmas for the input word without considering the provided POS information. If the lemma still cannot be identified, the original surface form is returned.

The CLEAR morphological analyzer [29] is also developed on top of the morphology functions of WordNet. In addition to the WordNet rules of detachment, it finds lemmas for some abbreviations (e.g.,*'re *→ *be*), generalizes ordinals (e.g., *21st *→ *$#ORD#$*), and shortens all numbers (e.g., *3.14 *→ *0.0*) in input words. The generalized lemmas have been demonstrated to be useful for some NLP tasks, for instance, dependency parsing [34]. However, since WordNet is not targeted at the biology domain, the performance of this and all WordNet-based lemmatizers on biomedical text suffers from its modest coverage of domain-specific terms [9,16,35,36].

The GENIA tagger [30] is a POS tagger specifically tuned for biomedical text. In addition to the POS tagging function, it also produces base forms for detected tokens. The morphological analysis focuses on four syntactic categories: noun, verb, adjective and adverb. The tagger maintains an exception list of irregular words, and a dictionary for both general English from WordNet [18] and biomedical language based on corpora such as GENIA [37] and PennBioIE [38]. A small set of rules is also used to heuristically handle tokens not encountered in the lexical resource. The dictionary is checked when rules require that the resulting transformed form be validated.

Similarly, TreeTagger [31] provides lemma information as part of POS tagging. However, since its lemmatization process solely relies on lexicon lookup, TreeTagger fails to retrieve lemmas for input words that are not recorded in the lexicon. Moreover, its ability to identify lemmas in the biology domain is restricted by the limited domain-specific coverage of the internal lexicon, despite having been applied in biomedical research [39]. A limitation of both the GENIA tagger and TreeTagger for lemmatization is that the lemmatization function is not separable from the POS tagging, and therefore cannot be used with a distinct tagging or parsing tool.

Norm [32] and LuiNorm [33] are lexical programs which normalize words in biomedical text using the Specialist lexicon [21]. Uninflected forms are generated using the Specialist lexicon directly if words appear in it; otherwise they are generated algorithmically [32]. Since the ultimate goal of these programs is to map normalized words to entries in the UMLS Metathesaurus [22], the normalization process additionally involves stripping possessives and diacritics, replacing punctuation with spaces, removing stop words, splitting ligatures, etc. [21]. Therefore, the resulting normalized form may differ substantially from lemmas obtained from other lemmatizers. POS information is not considered in the normalization process. When a form could be an inflection of more than one base form, Norm returns multiple base forms. In contrast, LuiNorm returns a single uninflected output for any input as it involves a process called canonicalization [33], which maintains a one-to-one correspondence between an input term and an output lemma even for ambiguously inflected input terms.

MorphAdorner [24] is a text analysis toolkit for general English, which consists of text processing components such as a sentence splitter, lemmatizer and POS tagger, and has been actively used in the Monk project [40]. Compared to other tools, the MorphAdorner lemmatizer maintains a word lexicon, a list of irregular forms and a set of rules for detachment, and makes use of them sequentially. Once a lemma is returned from any of the resources, the lemmatization process is complete. The BioLemmatizer tool we present is developed on top of the MorphAdorner lemmatizer, and has extended it in different aspects to cater to the needs of the biomedical domain. The lemmatization process of MorphAdorner will be discussed in more detail in the Methods section.

Unlike tools in which an explicit lexicon is actively maintained, *morpha *[17] is primarily a rule-based morphological analyzer. It comprises a set of approximately 1,400 rules, ranging from general rules that express morphological generalizations to specific rules that deal with a list of exceptions for irregular words. The rules are acquired semi-automatically from several large corpora and machine-readable dictionaries while the exception list is prepared from WordNet, containing about 5,000 verbs and 6,000 nouns [17]. *morpha *first checks the specific rules for an input word, and hands it over to the general rules if the word is not irregularly inflected. *morpha *has been incorporated into text mining systems in the recent biomedical research [2,14,15]. Although the lemmatization performance of *morpha *is not evaluated separately in these publications, some errors are expected since it was developed for general English morphology only.

## Results and discussion

We utilized three different biomedical datasets for evaluation of the BioLemmatizer, and compare the performance of this tool to the eight existing lemmatization tools introduced in Related Work. Next, we measure the individual contribution of each of the resources integrated in the BioLemmatizer. Furthermore, we describe in detail one practical application of the BioLemmatizer for the quality assurance of the CRAFT corpus [26,27]. Finally, we demonstrated how the BioLemmatizer contributes to accuracy improvement of an information extraction task [13] when it is used as a component in a biomedical text mining system [8,9] as compared to existing lemmatizers.

### Datasets

For evaluation of the BioLemmatizer, we require a corpus that both covers the domain of biomedicine and provides carefully curated lemma annotations. However, while most publicly available biomedical corpora contain gold annotations for tasks such as sentence segmentation, tokenization, POS tagging and entity identification [41,42], few are annotated with lemma information. To the best of our knowledge, the only biomedical corpus annotated with lemmas is the linguistically enriched version of the *LLL05 *challenge task corpus [43]. However, this is a fairly small dataset that contains only 141 sentences extracted from PubMed abstracts on the bacterium *Bacillus subtilis*. Specifically, there are only about 920 unique annotated pairs of (word, lemma). More importantly, the Part-of-Speech information is not provided in the corpus along with each annotated (word, lemma) pair. This makes the corpus less than ideal for evaluating the BioLemmatizer, since we would like to consider lemmatization performance in the situation when the word usage (POS tag) is clear, the normal use case in which a lemmatizer would be applied. However, we will report results on this corpus in combination with automated POS tagging below.

While it has been stated that all nouns and verbs in the GENIA corpus [37] have been lemmatized [44], the lemma information is not found in the public release of that corpus. Following up on this, we learned that the lemmas referred to in the original publication are not in fact manually curated but rather automatically generated by *morpha *[17], and that they are not available to the public (personal communication, Jin-Dong Kim).

We therefore prepared our own datasets to evaluate the performance of the BioLemmatizer. We have created the datasets based on two independent resources: the CRAFT corpus [26,27] and the Oxford English Dictionary [45].

The CRAFT corpus is a collection of 97 full-text open-access biomedical journal articles that have been used as evidential sources for Gene Ontology (GO) [46] annotations of genes and gene products of the laboratory mouse [26,47,48]. The corpus has been richly annotated both syntactically and semantically, and is provided as a community resource for the development of advanced BioNLP systems. The syntactic annotation includes sentence segmentation, tokenization, POS tagging, tree-banking and coreference linking. The CRAFT corpus has been under development by our group and our collaborators for the past three years and will soon be released to the public at *http://bionlp-corpora.sourceforge.net/CRAFT/index.shtml*.

We used the development subset of the CRAFT corpus, containing 7 full-text articles, as the basis for our first evaluation. We refer to it as **the CRAFT set**. Although curated annotation of lemmatization is not provided in CRAFT, we attempted to acquire lemma annotations for the CRAFT set semi-automatically. First, we ran all nine lemmatization tools, including the BioLemmatizer and the eight tools described in Related Work, against the CRAFT set. We built a "silver standard" based on the consensus of the lemma annotations produced by these lemmatization tools. The idea of the "silver standard" was first proposed in the CALBC challenge [49] to provide an annotation solution for large-scale corpora by making use of the harmonization of annotations from different systems. Next, annotation disagreements across the lemmatizers were manually resolved to form a "gold standard". Consensus among the tools was high and therefore only a modest amount of manual work was required to generate the gold standard.

We prepared our second dataset using the Oxford English Dictionary, which is known to have broad coverage of biomedical terminology. Most domain-specific entries in the OED are associated with a category label. Using 28 categories that we consider relevant to the biomedical domain, we collected a list of 11,269 OED entries including adjectives, adverbs, nouns and verbs. Inflected forms are recorded in the OED entries for nouns but not for the other parts of speech. Therefore, we focused on nouns and extracted a candidate list in which nouns are either provided with their irregularly inflected suffixes or marked as "noun plural" meaning that the singular and the plural forms of the noun are same. We then manually added the plural forms for candidate entries, and eventually obtained a list of 808 nouns together with their plurals, POS tags and OED categories, which we call **the OED set**. Table 1 lists the 28 OED categories considered in this work.

**Table 1:** OED categories related to biomedicine

Animal Physiology	Bacteriology	Biochemistry	Biology
Botany	Cytology	Embryology	Genetics

Geomorphology	Haematology	Immunology	Marine Biology

Medicine	Microbiology	Morphology	Old Medicine

Palaeobotany	Palaeontolgy	Palaeontology	Pathology

Physiological	Physiology	Pisciculture	Plant Physiology

Veg. Physiolology	Veterinary Medicine	Veterinary Science	Zoology

The gold annotation for POS tagging is not always available in real-world applications, for instance, when the BioLemmatizer is integrated into a text mining pipeline. In addition to our own datasets, therefore, we further prepared a third dataset by enriching the *LLL05 *corpus with automatically generated POS information. The POS tags were obtained from the GENIA tagger, which reports a 98.26% tagging accuracy on biomedical text [30]. Due to various word usage scenarios, the original *LLL05 *corpus was thus extended into 934 unique annotated triplets of (word, POS, lemma). We refer to this dataset as **the LLL05 set**, and it is intended to test the performance of the BioLemmatizer when POS errors potentially occur.

### Evaluation of BioLemmatizer on the CRAFT set

The CRAFT set contains a total of 67,653 tokens. Among them, there are 6,775 unique (token, POS) pairs. Because the various lemmatization tools are not consistent in their treatment of adverbs, including adverbs will prevent us from building a large consensus set. Therefore, we excluded all adverbs in the CRAFT set from the silver standard creation process, and evaluated them separately (results below). This left 6,441 unique (token, POS) pairs to serve as the test input, 95.07% of the original pairs.

Table 2 presents both the consensus and the disagreement of the lemmatization across different combinations of nine lemmatization tools including the BioLemmatizer, the WordNet lemmatizer [28], the CLEAR morphological analyzer [29], the GENIA tagger [30], TreeTagger [31], Norm [32], LuiNorm [33], MorphAdorner [24] and *morpha *[17]. The consensus rate among all nine tools was only about 71%, leaving a large number of disagreements. Since the CLEAR morphological analyzer generalizes ordinals and shortens all numbers in input words, the lemmas it produces often differ from those from the other tools. Furthermore, it is built using WordNet and is largely redundant with the WordNet lemmatizer for other terms. We therefore excluded the CLEAR morphological analyzer from the analysis, with an improved consensus rate among the eight remaining tools of 80%. We then removed Norm and LuiNorm from consideration due to their normalization steps, which also result in the production of substantially different lemma forms. The consensus rate across the remaining six tools exceeds 91%, leaving only a small set of disagreements.

**Table 2:** Consensus and disagreement of annotations across lemmatization tools

	Consensus (No.)	Percentage	Disagreement (No.)	Percentage
All 9 tools	4559	70.78%	1882	29.22%

8 tools				

(exclude CLEAR)	5207	80.84%	1234	19.16%

6 tools				

(further exclude Norm and LuiNorm)	5862	91.01%	579	8.99%

The consensus set of 5,862 lemma annotations, representing agreement between the BioLemmatizer, the WordNet lemmatizer [28], the GENIA tagger [30], TreeTagger [31], MorphAdorner [24] and *morpha *[17], are used as a "silver standard". Since some of these six tools share lexical resources and parts of rule sets, they might be inclined to make the same lemmatization mistakes. Therefore, we randomly selected 600 instances from the silver standard, about 10% of the total annotations, and manually examined them. The evaluation showed that the lemmatization accuracy is 100% on the random sample set, confirming that the silver standard we built is reliable.

The remaining 579 annotations were carefully manually reviewed to establish a "gold standard". One source of differences was spelling variation between British and American forms. The BioLexicon [19,20] uses British spelling in its lemma forms, e.g., *acetylise *and *harbour*, and therefore such forms are produced by the BioLemmatizer, while most of the other tools produce lemmas with American spellings. We therefore augmented our gold standard to allow both British and American spelling variants. Table 3 compares the performance of the BioLemmatizer with that of the other eight lemmatizers on the 579 gold lemmas in the CRAFT set. We employed the evaluation metrics of Precision, Recall and F-score to measure the performance of each lemmatizer rather than strict accuracy, because some tools, such as Norm and the WordNet lemmatizer, may return multiple lemmas for an input word, and some tools may not produce an output for every input (MorphAdorner and LuiNorm). Precision and Recall will be identical for the tools that always produce a single output lemma for each input.

**Table 3:** Lemmatization performance comparison of lemmatization tools on CRAFT set

	Recall	Precision	F-score
**BioLemmatizer**	**96.37% **(558/579)	**96.37% **(558/579)	**96.37%**

MorphAdorner	81.87% (474/579)	82.29% (474/576)	82.08%

*morpha*	72.71% (421/579)	72.71% (421/579)	72.71%

CLEAR	72.37% (419/579)	72.37% (419/579)	72.37%

WordNet	74.27% (430/579)	70.03% (430/614)	72.09%

GENIA Tagger	72.02% (417/579)	72.02% (417/579)	72.02%

Norm	83.25% (482/579)	59.36% (482/812)	69.30%

LuiNorm	62.18% (360/579)	62.50% (360/576)	62.34%

TreeTagger	50.78% (294/579)	50.78% (294/579)	50.78%

The data show that the BioLemmatizer outperformed the other tools we tested in lemmatizing biomedical texts by quite a large margin. The MorphAdorner lemmatizer achieved the second highest performance in both Precision and F-score, indicating that it generalizes well from the general English to the biology domain. Our extensions of the MorphAdorner system for the BioLemmatizer resulted in an improvement on biomedical text of greater than 14% in F-score. The Norm tool obtained the second highest recall, however, the overall F-score is only in the 60% range due to its disregard of POS information and the resulting generation of false positives.

We performed error analysis on the 21 false positive lemmas the BioLemmatizer produced, and identified four major causes of errors.

(1) **Errors in the lexicon**: We observed that 8 false positive lemmas resulted from the errors in the BioLemmatizer lexicon. Most of these errors derived from the BioLexicon [19,20]. For instances,

*biphenyls *(BioLexicon:LM_CHEBI_CHEBI:22888_1),

*Nematodes *(BioLexicon:LM_NcbiT_NCBITaxon:333870_1),

*longer *(BioLexicon:LM_MANCU_V1MPL10720_1),

*worse *(BioLexicon:LM_MANCU_V1MPL2636_1), and

*Fungi *(BioLexicon:LM_NcbiT_NCBITaxon:4751_1) were incorrectly recorded in the BioLexicon as lemmas instead of inflected forms. In addition, the comparative and superlative forms of some adjectives, and some plural nouns are incorrectly considered lemmas in the GENIA tagger resources [30] such as *biggest, highest, older, lesser, hearts, organs *and *primates*.

(2) **Errors in lemmatization rules**: One detachment rule, derived from the original MorphAdorner rule set, that was applied to terms not found in the lexicon contributed to 6 false positive lemmas on the CRAFT set. The inputs (immunolabeled, VBN) and (radiolabeled, VBN) are transformed into *immunolabele *and *radiolabele *by the detachment rule that generally requires removal of the ending character "d" of input verbs to produce the corresponding lemmas. However, in these cases this transformation results in a string that is not a valid English word.

(3) **Incorrect input POS information**: Furthermore, we noticed that some lemmatization errors are caused by errors derived from POS tag errors in the source CRAFT annotation. For instance, (Biosystems, NNP), (Neomarkers, NNP) and (Biosciences, NNP) return *Biosystems, Neomarkers *and *Biosciences *as lemmas instead of their correct forms *Biosystem, Neomarker *and *Bioscience*. The correct forms would have been obtained if the input had used the correct POS tag NNPS. They were missed because these input terms are not recorded in the lexicon, and the subsequent rule component determines that as singular proper nouns (NNP) these terms should not be lemmatized. Therefore, their original surface forms are returned as lemmas, leading to errors in 5 cases.

(4) **Errors in abbreviation handling**: Inherited from MorphAdorner, the BioLemmatizer finds lemmas for some abbreviations and symbols, such as producing "and" for the input '&'. However, in the biology domain, the expansion of a general English abbreviation sometimes produces errors. For instance, returning "saint" for '*St*.' may be inappropriate. It would be more accurate to return the original surface form of the abbreviations appearing in biomedical texts.

Comparing the BioLemmatizer results to results from the other tools also revealed that our hierarchical lexicon search strategy (see Methods) allows the BioLemmatizer to retrieve correct lemmas for many input terms even in conjunction with inaccurate POS information, where other tools are unable to produce a correct lemma. For instance, *anlagen *is wrongly tagged as singular noun in the CRAFT set. Given the input (anlagen, NN), the BioLemmatizer is the only one of the nine lemmatization tools that retrieves the correct lemma *anlage*. It is also the only tool that can discover the lemma *spermatogonium *for the input (spermatogonia, NN) among the eight tools that contain an explicit lexicon component. Similarly, the BioLemmatizer also found correct lemmas for erroneous inputs (Laboratories, NNP), (Products, NNP), (retinas, NN), (odds, NNS), etc. By taking advantage of this novel search strategy, the BioLemmatizer lexicon lookup is not restricted to the inaccurate input POS, and is able to discover the correct lemma through a broader, yet hierarchically constrained, search.

In addition, we evaluated the adverbs in the CRAFT set independently. Different tools have different conventions for handling adverbs, in some cases mapping them to their derivationally related adjectives and in some cases leaving them unchanged. These different conventions would lead to unfair penalties in the evaluation. We therefore only evaluated the BioLemmatizer performance, through manual review of the lemmas. A total of 1821 adverbs occur in the CRAFT set. Among them, there are 334 unique (adverb, POS) pairs. The BioLemmatizer attempts to transform adverbs to their derivationally related adjectives based on that functionality in the underlying MorphAdorner lemmatizer, for instances, "evolutionarily" to "evolutionary", "homologously" to "homologous", "microscopically" to "microscopical", and "transcriptionally" to "transcriptional". For the comparative and superlative forms of adverbs, the BioLemmatizer returns their base form adverb, e.g., "best" to "well" and "most" to "much". The original adverbs are returned if their related adjective forms do not exist, such as "sometimes", "nevertheless", "afterwards" and "elsewhere". In addition, for some adverbs, they share the same form with their corresponding adjectives, e.g., "downstream", "upstream", "likely" and "weekly". Our evaluation detected only two incorrect lemmas in the 334 unique pairs, "strikingly" and "accordingly". The BioLemmatizer returned "strike" and "accord" instead of the correct lemmas "striking" and "according". This was due to lexical entries inherited from MorphAdorner that relate these adverbs to their source verbs.

Considering the gold annotation together with the adverb set, the BioLemmatizer produces in total only 23 false positive lemmas for the 913 unique tokens that have lemmatization discrepancies among 9 tools, leading to an overall lemmatization accuracy of 97.5% on the fully reviewed sections of the CRAFT set.

### Evaluation of BioLemmatizer on the OED set

Table 4 demonstrates the performance of the BioLemmatizer in comparison with that of the other eight lemmatizers on the 808 nouns in the OED gold standard. Compared to the CRAFT set, the OED set is more difficult to lemmatize for two reasons. First, it contains only domain-specific terms while the CRAFT set contains a mix of domain-specific and general language words. Second, by design it only contains terms with an irregularly inflected plural form. In fact, the plurals of some entries on the list are noted as "rare" by OED.

**Table 4:** Lemmatization performance comparison of lemmatization tools on OED set

	Recall	Precision	F-score
**BioLemmatizer**	81.56% (659/808)	**81.56% **(659/808)	**81.56**%

*morpha*	75.74% (612/808)	75.74% (612/808)	75.74%

LuiNorm	73.02% (590/808)	73.02% (590/808)	73.02%

Norm	**85.64% **(692/808)	61.18% (692/1131)	71.37%

CLEAR	62.50% (505/808)	62.50% (505/808)	62.50%

MorphAdorner	55.45% (448/808)	55.45% (448/808)	55.45%

WordNet	56.56% (457/808)	54.21% (457/843)	55.36%

TreeTagger	53.96% (436/808)	53.96% (436/808)	53.96%

GENIA Tagger	49.01% (396/808)	49.01% (396/808)	49.01%

The BioLemmatizer achieved the highest Precision and F-score among all the tools. In notable contrast with the results on the CRAFT set, it produced 149 false positive lemmas on this data, confirming that the OED set is a much harder test set. The *morpha *tool obtained the second highest performance on the OED set and the third highest performance on the CRAFT set at the level of 70% in both recall and F-score, indicating that the rule-based lemmatization is stable when generalized from the general English to the biology domain. Norm achieves the best lemmatization recall by correctly identifying the most gold lemmas of the OED set, 33 more than the BioLemmatizer. We believe that this is because one of the reference sources used by the Specialist lexicon upon which Norm is developed is the Oxford Advanced Learner's Dictionary [21]. Therefore, most entries in the OED set are likely to have been incorporated directly into the Specialist lexicon.

### Evaluation of BioLemmatizer on the LLL05 set

We demonstrated the performance of the BioLemmatizer as compared to that of the other eight lemmatizers on the LLL05 set in terms of the gold lemma annotation in the 934 unique triplets. The results are presented in Table 5. Since the treatment of adverbs is not consistent across the various lemmatization tools, as discussed previously, we considered both inflectionally and derivationally derived lemmas of adverbs as correct in this evaluation, e.g. both "predominantly" or "predominant" are considered correct for "predominantly".

**Table 5:** Lemmatization performance comparison of lemmatization tools on LLL05 set

	Recall	Precision	F-score
**BioLemmatizer**	**97.64% **(912/934)	**97.64% **(912/934)	**97.64**%

MorphAdorner	97.22% (908/934)	97.22% (908/934)	97.22%

GENIA Tagger	96.79% (904/934)	96.79% (904/934)	96.79%

*morpha*	96.36% (900/934)	96.36% (900/934)	96.36%

TreeTagger	96.25% (899/934)	96.25% (899/934)	96.25%

WordNet	96.90% (905/934)	95.36% (905/949)	96.12%

CLEAR	93.36% (872/934)	93.36% (872/934)	93.36%

LuiNorm	84.90% (793/934)	85.92% (793/923)	85.41%

Norm	90.79% (848/934)	79.55% (848/1066)	84.80%

The BioLemmatizer achieved a 97.64% F-score and led the lemmatization performance in every evaluation metric among all the tools. However, it also produced 22 errors according to *LLL05*'s gold lemmas. We closely examined these lemmas and concluded that the errors exclusively come from two sources: incorrect or inconsistent lemma annotation, and task-specific normalizations. First, although it is stated that all lemmas in the *LLL05 *corpus have been manually verified [43], we observed some incorrect and inconsistent instances in the annotation with respect to our annotation guidelines, detailed in Table 6.

**Table 6:** Incorrect and inconsistent instances in LLL05 set

	Token	Generated POS	BioLemmatizer lemma	LLL05 gold lemma
1	predominants	NNS	predominant	predominants

2	coding	NN	coding	code

3	Most	JJS	many	most

4	primer	NN	primer	prime

5	directed	JJ	directed	direct

6	might	MD	may	might

7	located	JJ	located	locate

8	least	JJS	little	least

9	more	RBR	much	more

We further validated the POS tags of these cases generated by the GENIA tagger, since the POS has a decisive impact on the resulting lemma. For instances, the gold annotation would be correct for "directed" and "located" if the POS is "VBD" or "VBN", and for "coding" if the POS is "VBG". Our examination confirmed that the POS tags generated in these cases are all correct. Specifically, the sentence phrases in which "located" and "coding" occur are: "located upstream of the sspE locus" and "the dnaE coding region". Moreover, the lemma for "primer" is not consistently annotated as both "primer" and "prime" appear in the gold annotation. In addition, "predominant" is commonly used as adjective in general English but tagged as noun by the tagger. The sentence context "the former transcripts being predominants at the exponential growth phase" where "predominants" appears, clearly demonstrates the different usage of words in the biomedical text, highlighting the need of a domain-specific lemmatization tool.

Furthermore, the *LLL05 *corpus was originally designed for the Genic Interaction Extraction Challenge [43]. According to the task specification, the tokens involving named entities have been normalized into reference entities. For instance, "ykvD" was normalized into "kinD" since "kinD" was curated as the reference named entity for "ykvD" because of the original text "In vivo studies of the activity of four of the kinases, KinA, KinC, KinD (ykvD) and KinE (ykrQ)......". Also, "B." was transformed into its full form "Bacillus" while "fulfill" was considered lemma for its spelling variant "fulfil". In fact, these task-specific normalizations required abilities beyond the canonical lemmatization, and therefore contributed to the remaining 13 evaluation errors, presented in Table 7.

**Table 7:** Task-specific normalization instances in LLL05 set

	Token	Generated POS	BioLemmatizer lemma	LLL05 gold lemma
1	sigmaG	NN	sigmaG	sigG

2	sigmaK	NN	sigmaK	sigK

3	sigmaE	NN	sigmaE	sigE

4	sigmaA	NN	sigmaA	sigA

5	sigmaD	NN	sigmaD	sigD

6	sigmaF	NN	sigmaF	sigF

7	sigmaL	NN	sigmaL	sigL

8	sigmaB	NN	sigmaB	sigB

9	sigmaH	NN	sigmaH	sigH

10	ykvD	NN	ykvD	kinD

11	ykrQ	NN	ykrQ	kinE

12	B.	NNP	B.	Bacillus

13	fulfil	VB	fulfil	fulfill

After fixing incorrect or inconsistent instances and ignoring task-specific normalizations in the gold lemma annotation, Table 8 shows the performance of the tools on the updated LLL05 set. The BioLemmatizer was able to achieve a 100

**Table 8:** Lemmatization performance comparison of lemmatization tools on updated LLL05 set

	Recall	Precision	F-score
**BioLemmatizer**	**100.00% **(934/934)	**100.00% **(934/934)	**100.00**%

MorphAdorner	98.93% (924/934)	98.93% (924/934)	98.93%

GENIA Tagger	97.97% (915/934)	97.97% (915/934)	97.97%

*morpha*	97.75% (913/934)	97.75% (913/934)	97.75%

WordNet	98.18% (917/934)	96.63% (917/949)	97.40%

TreeTagger	96.68% (903/934)	96.68% (903/934)	96.68%

CLEAR	94.65% (884/934)	94.65% (884/934)	94.65%

LuiNorm	85.87% (802/934)	86.89% (802/923)	86.38%

Norm	91.86% (858/934)	80.49% (858/1066)	85.80%

### Evaluation of BioLemmatizer resources

In order to investigate the individual contribution of the lemmatization resources, we conducted experiments to evaluate the performance impact of different combinations of the BioLemmatizer resources. Table 9 presents the lemmatization performance of these combinations on both the silver and gold annotations of the CRAFT set. Table 10 presents the lemmatization performance of these combinations on the OED set.

**Table 9:** Lemmatization performance of the BioLemmatizer resources on CRAFT set

Silver Standard			
	**Recall**	**Precision**	**F-score**

Base (MorphAdorner lexicon)	94.37% (5532/5862)	94.16% (5532/5875)	94.26%

Base + GENIA	94.20% (5522/5862)	93.90% (5522/5881)	94.05%

Base + BioLexicon	98.41% (5769/5862)	98.23% (5769/5873)	98.32%

Entire Lexicon	98.60% (5780/5862)	98.42% (5780/5873)	98.51%

Rule Only	97.83% (5735/5862)	97.83% (5735/5862)	97.83%

Rule + Lexicon Validation	98.67% (5784/5862)	98.67% (5784/5862)	98.67%

**Gold Standard**			

	Recall	Precision	F-score

Base (MorphAdorner lexicon)	53.71% (311/579)	53.34% (311/583)	53.52%

Base + GENIA	62.69% (363/579)	61.95% (363/586)	62.32%

Base + BioLexicon	64.77% (375/579)	64.10% (375/585)	64.43%

Entire Lexicon	76.68% (444/579)	75.90% (444/585)	76.29%

Rule Only	85.84% (497/579)	85.84% (497/579)	85.84%

Rule + Lexicon Validation	90.85% (526/579)	90.85% (526/579)	90.85%

**Table 10:** Lemmatization performance of the BioLemmatizer resources on OED set

	Recall	Precision	F-score
Base (MorphAdorner lexicon)	53.34% (431/808)	53.34% (431/808)	53.34%

Base + GENIA	52.97% (428/808)	52.97% (428/808)	52.97%

Base + BioLexicon	54.08% (437/808)	54.08% (437/808)	54.08%

Entire Lexicon	54.21% (438/808)	54.21% (438/808)	54.21%

Rule Only	66.96% (541/808)	66.96% (541/808)	66.96%

Rule + Lexicon Validation	71.29% (576/808)	71.29% (576/808)	71.29%

We investigated the lemmatization performance impact of both the lexical resources and the rule resources of the BioLemmatizer. "Base" denotes the original lexicon resource from MorphAdorner, including both the word lexicon and the exception list of irregular words. "Base + GENIA" refers to the combination of the MorphAdorner lexical resource and the GENIA tagger resource. "Base + BioLexicon" represents the combination of the MorphAdorner lexical resource and the BioLexicon resources. "Entire Lexicon" refers to the BioLemmatizer lexicon. "Rule Only" indicates that the lemmatization process is only based on the application of the detachment rules of the BioLemmatizer, without any lexical information whatsoever. "Rule + Lexicon Validation" means that the lexicon validation constraint is enforced for the lemmatization rules for which this is relevant. The lexicon validation constraint requires that the produced lemma exists in the lexicon. This constraint compensates for the generation of invalid lemmas after application of some rules and allows the system to continue attempting rules until a valid lemma is produced. For instance, given that the word "appendixes" is not in the lexicon, a set of rules is applied to it sequentially. The first matched rule requires that the suffix of the word be converted from "xes" into "xis", and then results in a candidate lemma "appendixis". Since the lexicon validation constraint is enforced for this detachment rule, the system is notified that the resulting candidate lemma might be invalid since "appendixis" does not appear in the lexicon. Then, the system continues to attempt the next matched rule that asks to strip the ending "es", leading to the correct lemma "appendix" for "appendixes".

For all lexicon-based lemmatization experiments, if the (word, POS) combination cannot be found by the standard BioLemmatizer lexicon lookup procedure, we allow the lexicon to be checked again for the input word without using the provided POS information. This is a backup strategy, more permissive than the hierarchical search strategy, that maximizes the chance of finding the lemma in the lexicon for an input word. As a result, the lexicon-based lemmatization might produce more than one lemma for a given input word as lemmas corresponding to all possible parts of speech will be returned, resulting in some detrimental impact on the precision performance.

For the silver standard of the CRAFT set, the BioLexicon resource exhibits a more significant impact on the improvement of the lemmatization performance than the GENIA resource. However, the combination of the three source lexical resources yields the best performance, by a small margin. Enforcing the lexicon validation clearly improves the performance of the rule-based lemmatization, achieving performance comparable to the lexicon-based lemmatization by reducing false positive lemmas, further emphasizing the importance of the valuable lexical resources. When performing on the more difficult gold CRAFT set, the rule-based lemmatization outperforms even the entire lexicon-based lemmatization by a large margin. This indicates that the lemmatization rules can be quite reliable, and hence that the system can be expected to perform well even on novel vocabulary terms.

Table 10 presents the lemmatization performance of different combinations of the BioLemmatizer resources on the OED set. For this most difficult set, the combination of "Rule + Lexicon Validation" achieves a F-score of 71.3%, which is in fact better than most other tools on this data as shown in Table 4. The 4% increase in F-score of "Rule + Lexicon Validation" over "Rule Only" also confirms the important role of the lexicon. However, the gap in performance as compared to the full BioLemmatizer system on this data set (which achieved 81.6% F-score) demonstrates that making full use of both resources in the lemmatization process leads to the best performance.

After the experiments reported here, we collected all false positive lemmas we encountered, and we have fixed nearly all of them, either by adding an entry to the BioLemmatizer lexicon or by modifying the rules of detachment, in some cases adding the lexicon validation constraint. The gold lemma annotation of the OED set and both silver and gold standards of the CRAFT set have been made publicly available via http://biolemmatizer.sourceforge.net. In addition to the lemmatization tool that utilizes both lexical and detachment rule resources, we have also made both the best performing lexicon-based and rule-based lemmatization strategies accessible in the release of the BioLemmatizer to cater to various lemmatization scenarios.

### Quality assurance of the CRAFT corpus

In preparation for its upcoming final release, the CRAFT corpus [26,27] is being scrutinized to assure the quality of all manual syntactic and semantic annotations. The quality assurance process focuses on two aspects: annotation inconsistencies and annotation errors.

Annotation inconsistency refers to the degree of annotation disagreement among annotators. Annotation consistency is important for the coherence of the corpus, and to increase the utility of the corpus for the training of automatic systems [50]. If humans cannot agree on the annotations for certain cases, it is hard to imagine that any computational method could produce correct answers. Indeed, the way humans solve these problems is the reference criterion for the automatic assignment of annotations. Annotation errors refer to recognizing actual annotation mistakes, often during reconciliation of inconsistent annotations. When experimenting with the CRAFT set, we observed some inconsistent and incorrect Part-of-Speech annotations in the 7 full-text articles. Such problems are inevitable in any manually annotated corpus. Even for a widely used, high-quality corpus such as the GENIA corpus, the curated POS tag information is not always correct across the whole corpus [51]. Therefore, the main objective of the quality assurance is to minimize both inconsistencies and errors that occur in the annotation of the CRAFT corpus.

As we described above, in many cases the BioLemmatizer was able to retrieve correct lemmas for input nouns even supplied with an inaccurately tagged POS, due to the ability to search the lexicon hierarchically. We therefore decided to use the BioLemmatizer to help identify the potential POS tagging errors of nouns in the CRAFT corpus. The CRAFT corpus [26,27] has been divided into two portions: 70% of the corpus is prepared for the public release which contains 67 full-text articles while the remaining 30% (30 articles) is reserved for future blind evaluation purposes. Starting with the public release portion of CRAFT, we applied the BioLemmatizer to all tokens in the 67 articles, and pulled out a list of all the nominal tokens that met one of the following two criteria: (1) the input noun is identical to the lemma but the input POS is "NNS" or "NNPS" (potentially a singular noun incorrectly tagged as plural). (2) the input noun has a different form from the lemma but the input POS is "NN" or "NNP" (potentially a plural noun incorrectly tagged as singular). We consider that the POS assignment for these nouns is only potentially erroneous since for some nouns the singular and plural forms are identical, such as *series *and *species*. This list of potential errors was then reviewed by the annotators who are responsible for the syntactic annotation of the corpus. Eventually, out of the 1,299 candidate POS tagging errors automatically identified by the BioLemmatizer, 605 cases were confirmed, resulting in an error detection accuracy of 46.6%. The annotators were able to correct all the true tagging errors, such as (retinas, NN), (retina, NNS), (morulae, NN) and (papilla, NNS) in the official treebank annotation. The same quality assurance process for the 30% reserved portion of the CRAFT corpus has also been planned.

We also tested the processing speed of the BioLemmatizer. Discounting the time required to load the word lexicon of over 340,000 entries (a couple of seconds), the lemmatization process is very fast: the BioLemmatizer returns lemmas at a rate of more than 140 K per second when lemmatizing all 560,993 POS-tagged tokens in the public release portion of the CRAFT corpus, as measured on an iMac 3.6 GHz Dual-Core Intel Core i5 workstation. Since some of the evaluated tools such as the GENIA tagger and TreeTagger are not developed for lemmatization purpose but provide lemma information as part of the main function (e.g., POS tagging), a direct comparison of lemmatization speed among them is not straightforward. According to the published figures, however, this processing rate is comparable to other lemmatization tools such as *morpha *[17], even though a hierarchical search strategy has been employed in the BioLemmatizer.

### Contribution of BioLemmatizer to biomedical event extraction

The BioNLP'11 shared task (BioNLP-ST 2011) focused on automatically extracting semantic events involving genes or proteins in the biological literature across various sub-domains of molecular biology, such as binding events or post-translational modifications [13]. Automatic event extraction has a broad range of biological applications, ranging from support for the annotation of molecular pathways to the automatic enrichment of biological process databases [52]. It also facilitates the construction of complex conceptual networks since events can serve as participants in other events [53].

We participated in BioNLP-ST 2011 and proposed a novel subgraph matching-based approach [8,9] to tackle the GENIA event extraction (GE) task [54], and the Epigenetics and Post-translational Modifications (EPI) task [55], two main tasks of the shared task. Rules for detecting biological events are first automatically learned by identifying the key contextual dependencies from full syntactic parsing of annotated texts. Events are then recognized by searching for an isomorphism between dependency graphs of automatically learned event rules and complete sentences in the input texts. This process is treated as a subgraph matching problem, which corresponds to the search for a subgraph isomorphic to a rule graph within a sentence graph. The backtracking ability of our subgraph matching algorithm allows the event extraction process to recover from initial wrong matches and continue to proceed until the correct event is identified. We achieved a comparable precision with the top systems. However, our performance was affected by lower recall. Ranked by F-score, our performance ranked 9th and 6th in the two tasks respectively [13].

In addition to the above experiments where we have demonstrated the lemmatization accuracy figures of the BioLemmatizer on different biomedical datasets, we further demonstrated the contribution of the BioLemmatizer in the context of the event extraction task as compared to existing lemmatizers when the lemmatization tool is used as a component in our event extraction system [9]. Lemmatization is performed on every pair of node tokens to be matched to allow a node in the sentence graph to match with a node in the rule graph if their tokens share a same lemma. This evaluation can help researchers make the choice of incorporating a lemmatization component in their biomedical text mining system for complex information extraction tasks.

We conducted experiments on the GE task by applying the BioLemmatizer and five other tools that consistently achieve high performance across the evaluation datasets. We report our results on the development set of BioNLP-ST 2011, evaluated via the official online evaluation *http://www-tsujii.is.s.u-tokyo.ac.jp/GENIA/BioNLP-ST/GE/devel-eval.htm*. Table 11 presents the overall event extraction performance of our system using different lemmatization tools as component.

**Table 11:** Event extraction performance using various lemmatization tools on GE development set

	Recall	Precision	F-score
**BioLemmatizer**	**34.10% **(1106/3243)	58.70% (1106/1884)	**43.14**%

*morpha*	33.58% (1089/3243)	58.83% (1089/1851)	42.76%

GENIA Tagger	33.58% (1089/3243)	58.64% (1089/1857)	42.71%

MorphAdorner	33.52% (1087/3243)	58.57% (1087/1856)	42.64%

WordNet	33.21% (1077/3243)	**59.05% **(1077/1824)	42.51%

TreeTagger	33.09% (1073/3243)	58.89% (1073/1822)	42.37%

We observed that the BioLemmatizer helped to obtain the highest event extraction F-score by identifying 17 more events than the second highest performance achieved by *morpha*. In the context of our event extraction system, it indicates that the BioLemmatizer produces more correct lemmas than other tools for tokens in the biomedical text. This allows more event rules to match with unseen sentences, thus leading to more detected events. For instance, the domain-specific terms "post-translation" and "phosphorylation" are not recorded in the general English thesaurus WordNet, so lemmas of morphological variants of these terms cannot be correctly produced by the WordNet Lemmatizer. We also observed that the event extraction precision of the BioLemmatizer is lower than some tools such as TreeTagger. We attributed this to the fact that the precision of event rules is not always 100%. Although the high lemmatization accuracy from the BioLemmatizer helped to generate more potential matchings between rules and sentences, these matchings contain false positive events (see [9] for more details of our BioNLP-ST 2011 event extraction system).

Table 12 shows the detailed breakdown performance on *Binding *events, three regulation events such as *Positive_regulation, Negative_regulation *and *Regulation*, and five GE simple events including *Gene_expression, Transcription, Protein_catabolism, Phosphorylation *and *Localization*.

**Table 12:** Event extraction performance using various lemmatization tools on GE development set

Simple Events			
	**Recall**	**Precision**	**F-score**

**BioLemmatizer**	**59.21% **(656/1108)	77.82% (656/843)	**67.25%**

*morpha*	58.94% (653/1108)	77.92% (653/838)	67.11%

GENIA Tagger	58.84% (652/1108)	77.99% (652/836)	67.08%

WordNet	58.84% (652/1108)	77.90% (652/837)	67.04%

MorphAdorner	58.75% (651/1108)	77.87% (651/836)	66.98%

TreeTagger	58.30% (646/1108)	**78.21% **(646/826)	66.80%

**Binding Events**			

	Recall	Precision	F-score

TreeTagger	24.66% (92/373)	**44.66% **(92/206)	**31.78%**

**BioLemmatizer**	**24.93% **(93/373)	43.46% (93/214)	31.69%

*morpha*	24.93% (93/373)	43.46% (93/214)	31.69%

GENIA Tagger	24.93% (93/373)	43.46% (93/214)	31.69%

MorphAdorner	24.93% (93/373)	43.46% (93/214)	31.69%

WordNet	23.32% (87/373)	43.72% (87/199)	30.42%

**Regulation Events**			

	Recall	Precision	F-score

**BioLemmatizer**	**20.26% **(357/1762)	**43.17% **(357/827)	**27.58**%

*morpha*	19.47% (343/1762)	42.93% (343/799)	26.79%

GENIA Tagger	19.52% (344/1762)	42.63% (344/807)	26.78%

MorphAdorner	19.47% (343/1762)	42.56% (343/806)	26.71%

WordNet	19.18% (338/1762)	42.89% (338/788)	26.51%

TreeTagger	19.01% (335/1762)	42.41% (335/790)	26.25%

Table 12 further confirms that the recall improvement when using the BioLemmatizer mostly comes from the recognition of more complex, regulation events as compared to the other 5 tools. This indicates that authors tend to use a more biology-oriented terminology in the literature when describing complex events that often involve other events as the arguments, emphasizing the importance of this domain-specific lemmatization tool.

## Conclusions and future work

In this paper, we have described a domain-specific lemmatization tool, the BioLemmatizer, for the inflectional morphology processing of biological texts. The BioLemmatizer is developed based on the MorphAdorner general English lemmatizer, but extends that system in three major ways. First, a novel hierarchical search strategy is proposed to replace the original exact matching search method in order to maximize the chance of finding a lemma. Second, catering to the needs of the biological domain, the lemmatization resources are enriched through augmentation of the word lexicon and addition of detachment rules, based on two reputable biomedical resources. Third, the BioLemmatizer normalizes input terms that contain special Unicode characters to increase the opportunity of matching these terms in the lexicon (see the Methods section below).

We compared the BioLemmatizer to eight other existing lemmatization tools, using three datasets relevant to the biomedical domain created from the CRAFT corpus, the Oxford English Dictionary and the *LLL05 *corpus. The BioLemmatizer outperforms all other tools tested by a large margin, including its predecessor, the MorphAdorner lemmatizer. It achieves a lemmatization accuracy of 97.5% on the data derived from naturally occurring biomedical text. Furthermore, we investigated the individual contribution of each lemmatization resource in the BioLemmatizer, concluding that making full use of both lexicon and rule resources leads to the best performance. Moreover, we demonstrated a successful application of the BioLemmatizer for quality assurance of CRAFT corpus annotations. In the end, we evaluated the contribution of the BioLemmatizer to accuracy improvement of the biological event extraction task as compared to existing lemmatizers.

The BioLemmatizer is released as open source software to the BioNLP and text mining research communities, and is accessible from *http://biolemmatizer.sourceforge.net*. Also, the BioLemmatizer has been wrapped for use within UIMA pipelines [25]. All the experimental datasets, including the silver and gold annotations used for testing the BioLemmatizer, have also been made available. In addition, a Perl module of the BioLemmatizer is released on CPAN at *http://search.cpan.org/perldoc?Lingua::EN::BioLemmatizer*.

In our future work, we plan to extend the BioLemmatizer to address derivational morphology more fully. Nominalizations are used prevalently in biomedical text [56] as well as in the ontological resources of the biomedical domain such as the UMLS Metathesaurus [22] and Gene Ontology [46]. Analyzing the derivational morphology of biomedical texts would facilitate the mapping of terms from free text into these resources. For instance, verbs and adjectives such as "aspirate" and "hyperplastic" could respectively be mapped to the nominalized terms "Aspiration" and "Hyperplasia" in the UMLS Metathesaurus.

## Methods: Hierarchical search-based lemmatization

In this section, we first introduce the lemmatization process of MorphAdorner in more detail, since the BioLemmatizer is developed based on the MorphAdorner general English lemmatizer. We then describe the three major extensions made for the BioLemmatizer implementation, both with respect to the processing strategy and lemmatization resources. In the end, we illustrate the new lemmatization process of the BioLemmatizer as compared to that of the original lemmatizer.

### The MorphAdorner lemmatization process

**Figure 1:**
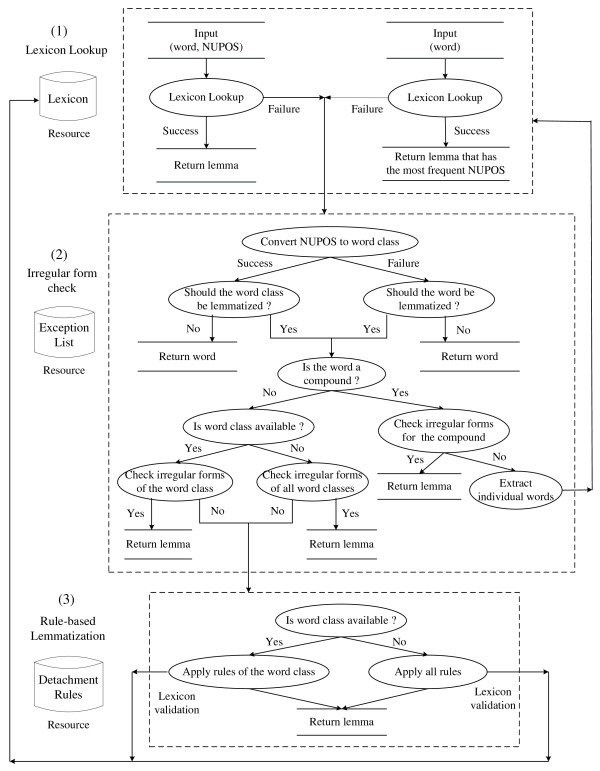
**The MorphAdorner lemmatization flow diagram**.

Figure 1 illustrates the overall lemmatization flow of MorphAdorner, which consists of three main steps: lexicon lookup, irregular form check and detachment rule-based lemmatization.

The word lexicon of MorphAdorner uses the NUPOS Part-of-Speech tagset [57], which is a much more fine-grained tagset than the prevailing Penn Treebank tagset. The current version of the NUPOS tagset contains 241 English parts of speech in comparison with 36 primary Penn Treebank POS tags [58]. Given an input word together with its NUPOS tag, i.e. the pair (word, NUPOS), MorphAdorner first checks if a lemma appears for the input pair in the word lexicon, and returns the specified lemma. When the pair is not found in the lexicon, MorphAdorner turns to the irregular form list and detachment rules. MorphAdorner maintains irregular forms and rules of detachment by grouping them into a number of major word classes, such as adjective, preposition, conjunction and verb. The input NUPOS tag is converted to one of the word classes. For instance, the NUPOS gerund tag *vvg *maps to the verb class. In this way, the subsequent lemmatization process is not tied to a specific, more fine-grained part of speech set. If the (word, word class) pair appears in the irregular form list, the lemmatizer returns the corresponding lemma specified in the list. Otherwise, the lemmatizer begins a series of rule matches for the converted word class. Each rule specifies an affix pattern to match and a replacement pattern which detaches the matching affix from the inflected form, and attaches any replacement characters to generate the final lemma form. The POS information for the input is optional for MorphAdorner. If the NUPOS is not provided, the lemmatizer either retrieves the lemma that has the most frequent part of speech from the lexicon according to the recorded word count information, or applies all rules across word classes. This can be useful when no POS tagger is available, however, the lemmatization accuracy will be seriously compromised.

Occasionally, multiple rules might be applicable to a given input. For instance, a verb ending in "ored" may correctly correspond to a lemma ending in "ore" (e.g., *implored *→ *implore*) or in "or" (e.g., *colored *→ *color*). To help disambiguate such cases, a lemmatization rule can specify that the resulting form must be validated by a known word list. For compound words, MorphAdorner attempts to split them into individual words at a logical point, assign a separate lemma to each word part using the regular lemmatization process, and concatenate them with a separator to form a compound lemma. For punctuation, symbols, singular nouns and foreign words determined by their characteristics or the provided NUPOS information, the original surface form is considered the lemma form. For some words, the lemma form can be ambiguous, for example, "axes" is the plural form of both "axe" and "axis". In this case, MorphAdorner returns one of the possible forms. However, this may not always be the correct form. In addition to inflectional morphology, derivational morphology is also explored in MorphAdorner, but is limited to the transformation of derived adverbs to their grammatically related adjectives.

### Extensions to MorphAdorner

The BioLemmatizer is built on top of the MorphAdorner lemmatizer, and has extended it in three major ways: hierarchical search of lexicon, enrichment of lemmatization resources, and normalization of special Unicode symbols.

#### Hierarchical search of lexicon

We have observed incorrect usage of technical terms in biomedical texts. For instance, many biology terms in plural form are incorrectly used as singular nouns in the literature, such as *anlagen *(pl.) → *anlage *(sg.), and *spermatogonia *(pl.) → *spermatogonium *(sg.). This leads to POS tagging errors. In practice, even for manually annotated, high-quality biomedical corpora such as CRAFT [26], linguistic annotators sometimes mistakenly assign a plural tag to a singular noun due to lack of domain-specific knowledge.

Incorrect POS tags have a direct, detrimental effect on the performance of lemmatizers. For instance, out of the eight lemmatizers described in Related Work, only *morpha *correctly returns "spermatogonium" for the input with an erroneous POS tag (spermatogonia, NN). There are two potential reasons that prohibit these tools from getting the correct result: the method used for searching the lexicon, and the criteria for application of the rules of detachment. The tools use an exact matching method to search for the (word, POS) combination. Therefore, even if "spermatogonium" exists in the lexicon, it will not be retrieved when the expected POS, NNS, is not provided in the input. In addition, the subsequent rule component of these tools also fails to produce the lemma because they largely depend on the input POS information to determine the subset of rules applicable to the input. Specifically, the input tag NN normally indicates that no lemmatization is necessary as the input is already a singular noun. Therefore the token "spermatogonia" will be left untouched even if the specific detachment rule to produce "spermatogonium" exists for the plural tag NNS.

**Figure 2:**
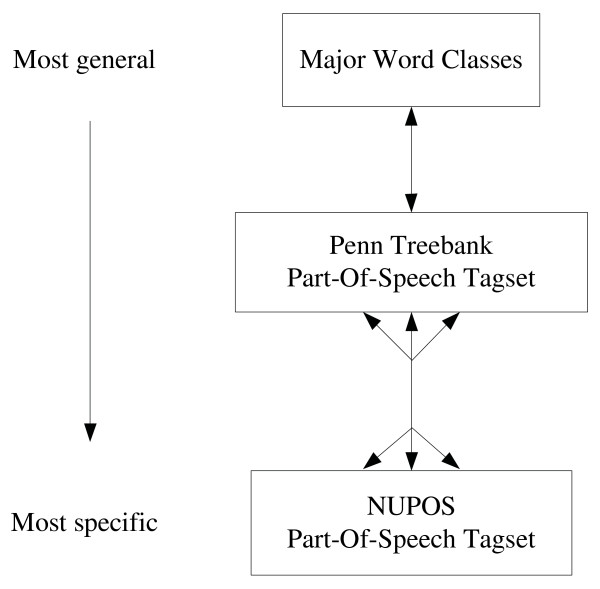
**The BioLemmatizer Part-Of-Speech search hierarchy**.

In order to address these potential POS-related lemmatization errors, we propose a novel hierarchical search strategy that utilizes the structure of a predefined POS hierarchy to enable controlled relaxation of a lexicon match. The hierarchy relates three tagsets: the fine-grained POS tags in the NUPOS tagset, the Penn Treebank tagset, and the MorphAdorner major word classes. The structure of the hierarchy appears in Figure 2. Integrating the three tagsets into a single hierarchy also allows us to take advantage of lexical resources defined in terms of any one of these individual tag sets, as we will explain further below.

Using this hierarchy, we can relax the strict POS match requirement during the lexicon lookup stage. Specifically, if the input (word, POS) pair is not found in the lexicon by exact match, a match using a structural "sibling" of the original POS will be attempted. In this hierarchy, for instance, the plural noun tag NNS is a sibling of the singular noun tag NN, so it specifically handles the problematic case introduced above. If this "sibling" search fails, the search will continue up or down the hierarchy to attempt a match with a more abstract POS or a more specific POS, depending on the tagset of the input POS and the availability of lexical resources tagged with this tagset. For instance, if an input POS is provided in terms of a Penn Treebank tag and no match to the word is found with that POS, the match will be attempted for that word in combination with each possible NUPOS tag which Penn Treebank tag is mapped to in the hierarchy. Conversely, if the input POS is a NUPOS tag, the search will be attempted with the corresponding more general Penn Treebank tag.

The premise of this strategy is that the lexicon lookup procedure always produces more reliable lemmas than the application of heuristic detachment rules. This is often true because most existing lexical resources have been manually curated by developers. In contrast, lemmatization rules are either hand-coded or semi-automatically acquired from a limited amount of training data, and they are always supplemented by an open set of exceptions for specific words. Therefore, the hierarchical search strategy maximizes the chance of finding a lemma in the lexicon for an input word by making the most of this valuable resource, and minimizes the potential incorrect application of heuristic rules.

The mapping between the Penn Treebank tagset and the NUPOS tagset is created manually based on our linguistic knowledge. Since the NUPOS tagset is more fine-grained than the Penn Treebank tagset, the 241 NUPOS tags are mapped into the 36 Penn Treebank tags. Due to the design of the NUPOS tagset, a few tags can be categorized into multiple Penn Treebank tags. For instance, the NUPOS tag *pc-acp *maps to two Penn Treebank tags, RP and TO. The multi-headed arrow in the figure indicates the many-to-many relationship between the two tagsets. The Penn Treebank tags are then mapped into the more general major word classes of MorphAdorner, forming a one-to-one relationship represented by the single-headed arrow.

Importing the Penn Treebank tagset into the hierarchy provides a number of advantages for lemmatization. First, it enables the search for an input word in resources tagged using either NUPOS and Penn Treebank tags. This facilitates the incorporation of other existing lexicons since the Penn Treebank tagset has been widely used in various lexical resources [37,38]. Moreover, it allows the BioLemmatizer to directly accept results from prevailing Penn Treebank tagset-based POS taggers as input, and still take full advantage of the internal lemmatization process of MorphAdorner by mapping Penn Treebank tags into NUPOS tags. Furthermore, the Penn Treebank tagset offers a more precise generalization level for the NUPOS tagset. Before traversing to the most general word classes, more accurate sibling tags for an NUPOS tag may be captured by the Penn Treebank tagset, leading to a more reliable lexicon lookup result. To the best of our knowledge, no previous efforts have been made to connect these two POS tagsets. The mapping files used in the predefined POS hierarchy are directly accessible at *http://biolemmatizer.sourceforge.net*.

In practice, the proposed hierarchical search strategy not only takes care of the wrong POS assignment between plural and single nouns, but also accommodates other lexical categories. For instance, it helps to regulate the frequent tagging errors between the past tense and the past participle of verbs. It also alleviates the need for a lexicon that covers a complete list of inflected forms of each verb.

#### Enrichment of lemmatization resources

The resources maintained by MorphAdorner are developed only for the morphological analysis of the general English. In order to tailor the BioLemmatizer to the biology domain, we have enriched the lemmatization resources, extending both the word lexicon and the detachment rules.

In addition to the core lexicon derived from MorphAdorner, the BioLemmatizer incorporates two domain-specific lexical resources for biology: the resources of the GENIA tagger [30], and morphological data in the BioLexicon database [19,20]. The morphological resources of the GENIA tagger are prepared based on WordNet [18], the GENIA corpus [37] and the PennBioIE corpus [38]. They are organized in terms of four syntactic categories: noun, verb, adjective and adverb, each associated with a dictionary and an exception list of irregular words. The GENIA corpus describes biological reactions concerning transcription factors in human blood cells, while the PennBioIE corpus covers the inhibition of the cytochrome P450 family of enzymes and molecular genetics of cancer. Therefore, the resources of the GENIA tagger focus on these domains of biomedical knowledge.

In contrast, the BioLexicon database is a large-scale terminological resource covering a much broader scope of semantic types, such as genes and proteins, chemical compounds, species, enzymes, diseases as well as various entities and concepts found in biological ontologies. It has been developed to address text mining requirements in the biomedical domain [20,59]. The BioLexicon also focuses on the same four categories: noun, verb, adjective and adverb. Each category accommodates both biomedical and general language words. The biomedical terms are either prepared from existing databases or automatically extracted from biomedical literature. In addition, different types of word variants are added into the BioLexicon, including inflectional, derivational, spelling and other variants. It has been demonstrated that the BioLexicon has a more in-depth coverage of vocabularies pertinent to the biology domain [19,20]. In this work, we focus on the lexical resources that contain lemmas, their parts of speech, and the corresponding inflectional forms. It is claimed that these resources of the BioLexicon have been manually curated [19,20]. The multi-word lemmas in the BioLexicon have been excluded, as we are only interested in the morphological analysis of individual words.

In order to incorporate these resources into the BioLemmatizer, they have been reorganized into a format conforming to the requirement of the MorphAdorner lexicon. That is, each lexicon entry must contain a single lemma followed by its inflected form for each possible part of speech. The original lemma is used if there is no inflectional variant of the lemma. Both biomedical resources utilize the Penn Treebank tagset.

In case that overlapping entries exist between the resources of the GENIA tagger and the BioLexicon, they are removed from the BioLexicon-derived lexicon. Some entries do also overlap with the base MorphAdorner lexicon, since these resources also include general language words used in biological text. However, they have no conflicts in the lexicon because of the different tagsets used, and therefore we can keep both in the lexicon. The final, integrated BioLemmatizer lexicon contains 346,965 entries, about 54% of these specific to the biomedical domain. The distribution of lexical entries in the BioLemmatizer lexicon is shown in Table 13.

**Table 13:** Distribution of sources for the BioLemmatizer lexicon

	Lexical Source	Domain of Focus	POS tagset	No. of Entries	**Perc**.
1	MorphAdorner	General English	NUPOS	161,166	46%

2	GENIA tagger	Biomedicine	Penn Treebank	68,990	20%

3	BioLexicon	Biomedicine	Penn Treebank	116,809	34%

Total	BioLemmatizer	Biomedicine	NUPOS, Penn Treebank	346,965	100%

To improve the heuristic handling of biomedical language for tokens not covered by the lexicon, we enriched the existing MorphAdorner rule set by adding rules derived from the GENIA tagger, as well as developing new detachment rules, for instances, the rule *mata *→ *ma *for *blastemata *→ *blastema*, the rule *ae *→ *a *for *amoebae *→ *amoeba*, and the rule *i *→ *us *for *lactobacilli *→ *lactobacillus*.

Application of some rules can result in an invalid lemma. We therefore added a lexicon validation constraint requiring that the produced lemma exists in the lexicon for these rules. If the constraint is not satisfied, the system continues by attempting application of other rules. Table 14 compares the number of rules of the BioLemmatizer with that of MorphAdorner in terms of different lexical categories, and also shows how many rules add the lexical lookup constraint.

**Table 14:** Lemmatization rule comparison between BioLemmatizer and MorphAdorner

	MorphAdorner	BioLemmatizer	MorphAdorner	BioLemmatizer
	**(Total)**	**(Total)**	**(Lexicon-enforced)**	**(Lexicon-enforced)**

Adjective	24	26	0	25

Adverb	3	3	0	0

Verb	163	165	6	11

Noun	10	22	0	6

Total	200	216	6	42

#### Normalization of special Unicode symbols

Biomedical texts, especially full text publications, contain a large diversity of Unicode characters [60]. For instance, special characters such as diacritics and ligatures are sometimes used in biomedical terms, such as *tænia *and *zoölogy*, with such forms often recorded as the only valid forms in the Oxford English Dictionary (OED) [45]. Terms containing such characters are generally normalized in the existing lexical resources, and so are notable to be matched to these resources in their naturally occurring form. To compensate for this, tools such as Norm [32] and LuiNorm [33] normalize diacritics and ligatures when mapping such terms into the UMLS Metathesaurus [22]. As a new functionality, the BioLemmatizer extends MorphAdorner to normalize the input terms that contain special Unicode, by stripping the diacritics and splitting the ligatures. For instances, *ö *is normalized to *o*, and *æ *is transformed into *ae*. This normalization process increases the chance of finding a lemma in the lexicon for input words that involve diacritics and ligatures.

### The BioLemmatizer lemmatization process

**Figure 3:**
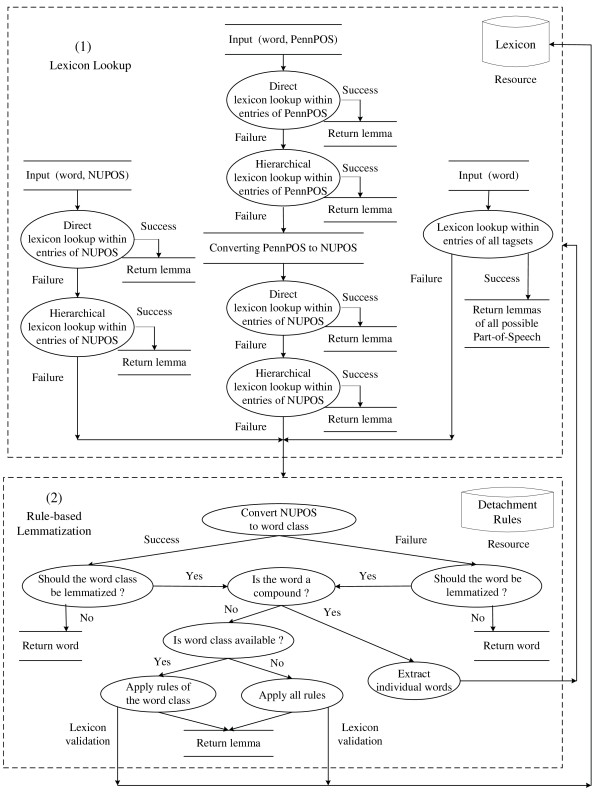
**The BioLemmatizer lemmatization flow diagram**.

Figure 3 presents the overall lemmatization flow of the BioLemmatizer, which consists of only two steps: lexicon lookup and rule-based lemmatization. Compared to MorphAdorner, the BioLemmatizer combines the steps of lexicon lookup and irregular form checkup. We do not consider the irregular form information to be distinct from other lexical information. Therefore, the exception list is integrated into the BioLemmatizer lexicon and can be accessed by the normal lexicon lookup procedure.

In addition to the MorphAdorner input of NUPOS tags, the BioLemmatizer also accepts as input a word together with its Penn Treebank tag. This combination is first checked by both the direct and hierarchical search strategies in the lexical resources of the tagset to which the input POS tag belongs. If the combination is not found, the input Penn Treebank tag is converted into a set of corresponding NUPOS tags based on the POS hierarchy. Then, the lexicon defined in terms of the NUPOS tagset is searched for the input word together with each of the possible NUPOS tags. In the current version of the BioLemmatizer, the search strategy across tagsets is only implemented for input that contains a Penn Treebank tag, since most of the prevailing POS taggers for BioNLP utilize the Penn Treebank tagset. However, generalizing input NUPOS tags to Penn Treebank tags to enable the search in resources tagged with both tagsets is straightforward, and can be implemented if there is demand.

When POS information is not given in the input, MorphAdorner returns only the lemma that has the most frequent part of speech. In contrast, the BioLemmatizer searches the entire lexicon and returns lemmas for all possible parts of speech, in termsof both tagsets represented in the lexicon. Our assumption is that without knowing the word context, the lemmatizer should return all possible lemmas and allow the user or calling application to resolve the ambiguities.

## Availability of supporting data

The executable jar file, the source code and the UIMA wrapper of the BioLemmatizer, along with the evaluation datasets used in the experiments described in this article, are available in the SourceForge SVN repository at *http://biolemmatizer.sourceforge.net*. In addition, a Perl module Lingua::En::BioLemmatizer is released on CPAN at *http://search.cpan.org/perldoc?Lingua::EN::BioLemmatizer*.

## Endnote

http://lexsrv3.nlm.nih.gov/SPECIALIST/index.html

## Competing interests

To the best knowledge of the authors, there are no competing interests.

## Authors' contributions

HL designed and implemented the BioLemmatizer, conducted all the experiments, and wrote the manuscript. TC provided knowledge of handling Unicode characters and the OED set for the in-depth evaluation of the tool, and developed the BioLemmatizer Perl module. WAB designed and implemented the UIMA wrapper for the BioLemmatizer, and contributed to the software engineering of the tool. KV conceived of the project, supervised the design of the BioLemmatizer, and contributed to the manuscript. All authors have read and approved this manuscript.

## References

[B1] Kanis J Skorkovská L Comparison of different lemmatization approaches through the means of information retrieval performance Proceedings of the 13th international conference on Text, speech and dialogue TSD'10 2010 93 100

[B2] Rinaldi F Schneider G Kaljurand K Clematide S Vachon T Romacker M OntoGene in BioCreative II.5 IEEE/ACM Trans Comput Biology Bioinform 2010 7 3 472 480 10.1109/TCBB.2010.50 20671319

[B3] Baeza-Yates R Ribeiro-Neto B Modern Information Retrieval 1999 Boston: Addison Wesley

[B4] Fuller M Zobel J Conflation-based Comparison of Stemming Algorithms Proceedings of the third Australian document computing symposium 1998 8 13

[B5] Porter MF An Algorithm for Suffix Stripping Program 1980 14 3 130 137 10.1108/eb046814

[B6] Paice CD Another stemmer SIGIR Forum 1990 24 56 61 10.1145/101306.101310

[B7] Korenius T Laurikkala J Järvelin K Juhola M Stemming and lemmatization in the clustering of finnish text documents Proceedings of the thirteenth ACM international conference on Information and knowledge management, CIKM'04 2004 625 633

[B8] Liu H Blouin C Keselj V Biological Event Extraction using Subgraph Matching Proceedings of the Fourth International Symposium on Semantic Mining in Biomedicine (SMBM 2010) 2010 Hinxton, Cambridgeshire, UK

[B9] Liu H Komandur R Verspoor K From Graphs to Events: A Subgraph Matching Approach for Information Extraction from Biomedical Text Proceedings of BioNLP Shared Task 2011 Workshop 2011 Portland, Oregon, USA: Association for Computational Linguistics 164 172

[B10] Ananiadou S Mcnaught J Text Mining for Biology And Biomedicine 2005 London: Artech House Publishers

[B11] Abacha AB Zweigenbaum P Medical Entity Recognition: A Comparison of Semantic and Statistical Methods Proceedings of BioNLP 2011 Workshop 2011 Portland, Oregon, USA: Association for Computational Linguistics 56 64

[B12] Chowdhury MFM Lavelli A Moschitti A A Study on Dependency Tree Kernels for Automatic Extraction of Protein-Protein Interaction Proceedings of BioNLP 2011 Workshop 2011 Portland, Oregon, USA: Association for Computational Linguistics 124 133

[B13] Kim JD Pyysalo S Ohta T Bossy R Nguyen N Tsujii J Overview of BioNLP Shared Task 2011 Proceedings of BioNLP Shared Task 2011 Workshop 2011 Portland, Oregon, USA: Association for Computational Linguistics 1 6

[B14] McClosky D Surdeanu M Manning C Event Extraction as Dependency Parsing for BioNLP 2011 Proceedings of BioNLP Shared Task 2011 Workshop 2011 Portland, Oregon, USA: Association for Computational Linguistics 41 45

[B15] Vlachos A Craven M Biomedical Event Extraction from Abstracts and Full Papers using Search-based Structured Prediction Proceedings of BioNLP Shared Task 2011 Workshop 2011 Portland, Oregon, USA: Association for Computational Linguistics 36 40

[B16] Ehsan Emadzadeh GG Azadeh Nikfarjam Double Layered Learning for Biological Event Extraction from Text Proceedings of BioNLP Shared Task 2011 Workshop 2011 Portland, Oregon, USA: Association for Computational Linguistics 153 154

[B17] Minnen G Carroll J Pearce D Applied morphological processing of English Natural Language Engineering 2001 7 207 223

[B18] Fellbaum C WordNet: An Electronic Lexical Database 1998 Cambridge: Bradford Books

[B19] Sasaki Y Montemagni S Pezik P Rebholz-Schuhmann D McNaught J Ananiadou S BioLexicon: A Lexical Resource for the Biology Domain Proceedings of the Third International Symposium on Semantic Mining in Biomedicine (SMBM 2008) 2008 Turku, Finland 109 116

[B20] Thompson P McNaught J Montemagni S Calzolari N del Gratta R Lee V Marchi S Monachini M Pezik P Quochi V Rupp C Sasaki Y Venturi G Rebholz-Schuhmann D Ananiadou S The BioLexicon: a large-scale terminological resource for biomedical text mining BMC Bioinformatics 2011 12 397 10.1186/1471-2105-12-397 21992002 PMC3228855

[B21] McCray AT Suresh Srinivasan ACB Lexical methods for managing variation in biomedical terminologies Proceedings of Annual Symposium on Computer Application in Medical Care 1994 235 239 PMC2247735 7949926

[B22] McCray AT Aronson AR Browne AC Rindflesch TC Razi A Srinivasan S UMLS knowledge for biomedical language processing Bull Med Libr Assoc 1993 81 2 184 94 8472004 PMC225761

[B23] MEDLINE Natonal Library of Medicine http://www.ncbi.nlm.nih.gov/PubMed Accessed in December 2011

[B24] Academic and Research Technologies, Northwestern University, MorphAdorner http://morphadorner.northwestern.edu/Accessed in December 2011

[B25] Ferrucci D Lally ADAM UIMA: an architectural approach to unstructured information processing in the corporate research environment Natural Language Engineering 2004 10 3-4 327 348 10.1017/S1351324904003523

[B26] Verspoor KM Cohen KB Lanfranchi A Warner C Johnson HL Roeder C Choi JD Funk C Malenkiy Y Eckert M Xue N B W A Jr Bada M Palmer M Hunter LE A corpus of full-text journal articles is a robust evaluation tool for revealing differences in performance of biomedical natural language processing tools BMC Bioinformatics 2011 in press 10.1186/1471-2105-13-207 PMC3483229 22901054

[B27] Bada M Eckert M Evans D Garcia K Shipley K Sitnikov D B W A Jr Cohen KB Verspoor K Blake JA Palmer M Hunter LE Concept Annotation in the CRAFT Corpus BMC Bioinformatics 2011 [Under review] 10.1186/1471-2105-13-161 PMC3476437 22776079

[B28] Pedersen T Banerjee S WordNet::Stem http://search.cpan.org/~tpederse/WordNet-Similarity-2.05/lib/WordNet/stem.pm Accessed in December 2011

[B29] Computational Language and EducAtion Research (CLEAR), Clear Morphological Analyzer http://code.google.com/p/clearparser/Accessed in December 2011

[B30] Tsuruoka Y Tateishi Y Kim JD Ohta T McNaught J Ananiadou S ichi Tsujii J Developing a Robust Part-of-Speech Tagger for Biomedical Text Advances in Informatics - 10th Panhellenic Conference on Informatics LNCS 3746 2005 382 392

[B31] Schmid H Probabilistic Part-of-Speech Tagging Using Decision Trees Proceedings of the International Conference on New Methods in Language Processing 1994 Manchester, UK

[B32] Lexical Systems Group, National Library of Medicine, Norm http://lexsrv3.nlm.nih.gov/LexSysGroup/Projects/lvg/2011/docs/userDoc/tools/norm.html Accessed in December 2011

[B33] Lexical Systems Group, National Library of Medicine, LuiNorm http://lexsrv3.nlm.nih.gov/LexSysGroup/Projects/lvg/2011/docs/userDoc/tools/luiNorm.html Accessed in December 2011

[B34] Choi JD Palmer M Getting the Most out of Transition-based Dependency Parsing Proceedings of the 49th Annual Meeting of the Association for Computational Linguistics: Human Language Technologies 2011 Portland, Oregon, USA: Association for Computational Linguistics 687 692

[B35] Bodenreider O Burgun A Mitchella JA Evaluation of WordNet as a source of lay knowledge for molecular biology and genetic diseases: a feasibility study Studies In Health Technology And Informatics 2003 95 379 384 14664016 PMC1893008

[B36] Burgun A Bodenreider O Comparing terms, concepts and semantic classes in WordNet and the Unified Medical Language System Proceedings of NAACL2011 Workshop, WordNet and Other Lexical Resources: Applications, Extensions and Customizations 2001 77 82

[B37] Kim JD Ohta T Teteisi Y Tsujii J GENIA corpus - a semantically annotated corpus for bio-textmining Bioinformatics 2003 19 suppl 1 i180 i182 10.1093/bioinformatics/btg1023 12855455

[B38] Kulick S Bies A Liberman M Mandel M McDonald R Palmer M Schein A Ungar L Winters S White P Integrated Annotation for Biomedical Information Extraction Proceedings of HLT/NAACL-2004 2004 Boston, Massachusetts, USA

[B39] Warnier P Nédellec C Sentence Filtering for BioNLP: Searching for Renaming Acts Proceedings of BioNLP Shared Task 2011 Workshop 2011 Portland, Oregon, USA: Association for Computational Linguistics 121 129

[B40] Kumar A MONK Project: Architecture Overview Proceedings of JCDL 2009 Workshop: Integrating Digital Library Content with Computational Tools and Services 2009 Austin, Texas, USA

[B41] Cohen KB Ogren PV Fox L Hunter L Corpus design for biomedical natural language processing ISMB'05: Proceedings of the ACL-ISMB Workshop on Linking Biological Literature, Ontologies and Databases 2005 Morristown, NJ, USA: Association for Computational Linguistics 38 45

[B42] Pyysalo S Ginter F Heimonen J Björne J Boberg J Järvinen J Salakoski T BioInfer: a corpus for information extraction in the biomedical domain BMC Bioinformatics 2007 8 1 50 10.1186/1471-2105-8-50 17291334 PMC1808065

[B43] Nédellec C Learning Language in Logic - Genic Interaction Extraction Challenge Proceedings of the Learning Language in Logic 2005 Workshop at the International Conference on Machine Learning 2005

[B44] Erjavec T Kim JD Ohta T Tateisi Y ichi Tsujii J Encoding biomedical resources in TEI: the case of the GENIA corpus Proceedings of the ACL 2003 workshop on Natural language processing in biomedicine 2003 13 Stroudsburg, PA, USA: Association for Computational Linguistics 97 104

[B45] Oxford English Dictionary Third http://www.oed.com/Accessed in December 2011

[B46] Gene Ontology Consortium Creating the gene ontology resource: design and implementation Genome Research 2001 11 8 1425 1433 10.1101/gr.180801 11483584 PMC311077

[B47] Bada M Hunter LE Eckert M Palmer M An overview of the CRAFT concept annotation guidelines Proceedings of the Fourth Linguistic Annotation Workshop 2010 207 211

[B48] Cohen KB Lanfranchi A Corvey W B W A Jr Roeder C Ogren PV Palmer M Hunter L Annotation of all coreference in biomedical text: Guideline selection and adaptation Proceedings of BioTxtM 2010: 2nd workshop on building and evaluating resources for biomedical text mining 2010 37 41

[B49] Rebholz-Schuhmann D Jimeno-Yepes A van Mulligen EM Kang N Kors JA Milward D Corbett P Buyko E Beisswanger E Hahn U Calbc Silver Standard Corpus J Bioinformatics and Computational Biology 2010 8 163 179 10.1142/S0219720010004562 20183881

[B50] Civit M Ageno A Navarro B Bufi N Marti MA Qualitative and Quantitative Analysis of Annotators' Agreement in the Development of Cast3LB Proceedings of Second Workshop on Treebanks and Linguistic Theories - TLT2003 2003

[B51] Liu H Blouin C Keselj V Sentence identification of biological interactions using PATRICIA tree generated patterns and genetic algorithm optimized parameters Data & Knowledge Engineering 2010 69 137 152 10.1016/j.datak.2009.09.002

[B52] Ananiadou S Pyysalo S Tsujii J Kell DB Event extraction for systems biology by text mining the literature Trends in Biotechnology 2010 28 7 381 390 10.1016/j.tibtech.2010.04.005 20570001

[B53] Hoffmann R Valencia A A Gene Network for Navigating the Literature Nature Genetics 2004 36 664 http://www.ihop-net.org/10.1038/ng0704-664 15226743

[B54] Kim JD Wang Y Takagi T Yonezawa A Overview of the Genia Event task in BioNLP Shared Task 2011 Proceedings of the BioNLP 2011 Workshop Companion Volume for Shared Task 2011 Portland, Oregon: Association for Computational Linguistics

[B55] Ohta T Pyysalo S Tsujii J Overview of the Epigenetics and Post-translational Modifications (EPI) task of BioNLP Shared Task 2011 Proceedings of the BioNLP 2011 Workshop Companion Volume for Shared Task 2011 Portland, Oregon: Association for Computational Linguistics

[B56] Cohen KB Palmer M Hunter L Nominalization and alternations in biomedical language PLoS ONE 2008 3 9 e3158 10.1371/journal.pone.0003158 18779866 PMC2527518

[B57] Mueller M NUPOS: A part of speech tag set for written English from Chaucer to the present 2009

[B58] Marcus MP Santorini B Marcinkiewicz MA Building a Large Annotated Corpus of English: The Penn Treebank Computational Linguistics 1993 19 2 313 330

[B59] Rebholz-Schuhmann D Pezik P Lee V Kim J Calzolari N Monachini M Montemagni S del Gratta R Marchi S Quochi V Ananiadou S McNaught J Sasaki Y BioLexicon: Towards a Reference Terminological Resource in the Biomedical Domain Proceedings of the of the 16th Annual International Conference on Intelligent Systems for Molecular Biology (ISMB-2008) 2008

[B60] Cohen KB Christiansen T Hunter LE Parenthetically Speaking: Classifying the Contents of Parentheses for Text Mining Proceedings of American Medical Informatics Association Fall Symposium 2011 PMC3243264 22195078

